# Long non-coding RNA PWRN4 associated with post-SVR hepatocellular carcinoma: a genome-wide association study

**DOI:** 10.1186/s40364-025-00832-9

**Published:** 2025-09-24

**Authors:** Goki Suda, Masaya Sugiyama, Hayato Hikita, Akira Nishio, Tomohide Tatsumi, Tetsuo Takehara, Miyako Murakawa, Mina Nakagawa, Yasuhiro Asahina, Masashi Mizokami, Tatsuhiko Kakisaka, Yuzuru Sakamoto, Akinobu Taketomi, Koji Miyanishi, Yoshiyuki Ueno, Hiroaki Haga, Shinya Maekawa, Nobuyuki Enomoto, Masayuki Kurosaki, Motoyuki Kohjima, Makoto Nakamuta, Yasuhito Tanaka, Yoshiya Yamamoto, Masaru Baba, Hisatoshi Hanamatsu, Jun-Ichi Furukawa, Masatsugu Ohara, Takashi Kitagataya, Naoki Kawagishi, Masato Nakai, Takuya Sho, Koji Ogawa, Naoya Sakamoto

**Affiliations:** 1 Department of Gastroenterology and Hepatology, Hokkaido University Graduate School of Medicine, North 15, West 7, Kita-ku, Sapporo, 060-8638 Hokkaido Japan; 2 Department of Viral Pathogenesis and Controls, National Institute of Global Health and Medicine, Japan Institute for Health Security, Ichikawa, Japan; 3 Department of Gastroenterology and Hepatology, Osaka University Graduate School of Medicine, Suita, Japan; 4 Department of Gastroenterology and Hepatology, Institute of Science Tokyo, Tokyo, Japan; 5 Cellular and Molecular Biotechnology Research Institute, National Institute of Advanced Industrial Science and Technology, Tsukuba, Japan; 6 Department of Gastroenterological Surgery I, Hokkaido University Graduate School of Medicine, Sapporo, Japan; 7 Department of Medical Oncology, Sapporo Medical University School of Medicine, Sapporo, Japan; 8 Department of Gastroenterology, Faculty of Medicine, Yamagata University, Yamagata, Japan; 9 Gastroenterology and Hepatology Department of Internal Medicine, Faculty of Medicine, University of Yamanashi, Yamanashi, Japan; 10 Department of Gastroenterology and Hepatology, Musashino Red Cross Hospital, Tokyo, Japan; 11 Department of Gastroenterology, NHO Kyushu Medical Center, Fukuoka, Japan; 12 Department of Gastroenterology and Hepatology, Graduate School of Medical Sciences, Kumamoto University, Kumamoto, Japan; 13 Department of Gastroenterology and Hepatology, Hakodate Municipal Hospital, Hakodate, Hakodate Japan; 14 Center for Gastroenterology and Hepatology, Japan Community Healthcare Organization Hokkaido Hospital, Sapporo, Japan; 15 Institute for Glyco-Core Research (iGCORE), Nagoya University, Nagoya, Japan

**Keywords:** Long non-coding RNA, Genome-wide association study, Single nucleotide polymorphism, Hepatocellular carcinoma, Hepatitis c

## Abstract

A subset of patients still develops hepatocellular carcinoma (HCC) even after eradication of the hepatitis-C virus (HCV) by anti-HCV treatment. We conducted a genome-wide association study (GWAS) to identify host genetic factors associated with HCC development following HCV eradication in Japan. In this GWAS (*n* = 517), the discovery cohort included 118 patients without HCC and 67 who developed HCC following HCV eradication with interferon-based therapy. A genome-wide scan for HCC-associated variants was conducted. An independent cohort of 274 patients without HCC and 58 patients with post-eradication HCC was used for replication. The effects of candidate gene variants were assessed clinically and through in vitro cellular assays. The GWAS identified significant variants associated with HCC development following HCV eradication, including rs4778350, located near the long non-coding RNA Prader-Willi non-protein coding RNA 4 (PWRN4) on chromosome 15. In the combined analysis, rs4778350 remained significantly associated with HCC, showing a high odds ratio of 5.86 (95% CI, 3.63–9.44). The frequency of the A allele in rs4778350 differs across ethnic populations. Multivariate analysis revealed that female sex, high platelet count, and higher serum albumin levels were associated with reduced HCC risk, while fibrosis stage F4 and the AA genotype of rs4778350 were linked to increased risk. The AA genotype of rs4778350 enhanced PWRN4 expression, promoting cell proliferation, migration, and invasion. These findings suggest a role for PWRN4 in hepatocarcinogenesis through its association with rs4778350 in patients achieving HCV eradication.

**Supplementary Information:**

The online version contains supplementary material available at 10.1186/s40364-025-00832-9.

**To the Editor**,

Although recently developed anti-HCV therapies achieve high rates of sustained virologic response (SVR) in patients with chronic hepatitis C [1, 2], hepatocellular carcinoma (HCC) may still develop after successful viral eradication [3, 4, 5, 6]. Traditional clinical predictors (e.g., advanced fibrosis, older age, male sex, diabetes, and high alpha-fetoprotein) only partially explain the risk of post-SVR HCC [4]. Although prior studies identified a single nucleotide polymorphism (SNP) associated with HCC after SVR (odds ratio ~ 2.4) [7], the findings did not reach genome-wide significance and lacked consistent replication. Thus, germline genetic factors contributing to HCC following HCV cure remain unclear.

We conducted a genome-wide association study (GWAS) involving Japanese patients from 15 institutions who had achieved SVR, to identify novel risk variants. The discovery (*n* = 185) and replication (*n* = 332) cohorts comprised 125 individuals who developed HCC ≥ 12 months after SVR and 392 who did not. The GWAS identified a significant association between rs4778350, located near the long non-coding RNA PWRN4 (Prader–Willi region non-protein coding RNA 4) on chromosome 15 (Supplementary Fig. S1a) and post-SVR HCC occurrence (Table 1). This SNP exceeded the genome-wide significance threshold (*p* = 9.8 × 10^–15^) and conferred a markedly increased risk, with a combined odds ratio of 5.9 in carriers of the risk allele (Table 1). The cumulative incidence of HCC following treatment differed significantly according to rs4778350 genotype (*p* < 0.001) (Fig. 1A), even among patients with or without advanced liver fibrosis (Fig. 1B, C). To the best of our knowledge, rs4778350 is the first genetic variant to be associated with post-SVR HCC development at genome-wide significance. Multivariate analysis showed that female sex (OR, 0.22; 95% CI, 0.12–0.41; *p* < 0.001), high platelet count (OR, 0.89; 95% CI, 0.80–0.99; *p* = 0.045), and higher serum albumin levels (OR, 0.33; 95% CI, 0.13–0.87; *p* = 0.026) were significantly associated with reduced HCC risk after HCV eradication (Fig. 1D). Conversely, fibrosis stage F4 (OR, 4.4; 95% CI, 2.0–9.6; *p* = 0.0154) and the AA genotype of rs4778350 (OR, 5.6; 95% CI, 2.8–11.0; *p* < 0.001) were independently associated with increased HCC risk following HCV eradication (Fig. 1D).

**Table 1:** Baseline characteristics and association of rs4778350 with hepatocellular carcinoma (HCC) occurrence after hepatitis C virus (HCV) eradication

Total number	Control	HCC occurrence	R-control	R-HCC occurrence	OR^*^ (95% CI) *P*-Value**
	118	67	274	58	
Patient characteristics					
Age (years) ^a^	59 (25–85)	62 (42–85)	60 (16–82)	58 (37–84)	–
> 65 years *n* (%)	27 (22.9%)	25 (37.3%)	75 (27.4%)	22 (34.4%)	–
Sex (male/female)	56/62	51/16	114/160	39/19	–
Baseline platelet count (´10^4^/µL) ^a^	17.0 (7.4–29.9)	12.7 (5.7–24.0)	17.0 (6.3–36.5)	12.7 (7.4–28.3)	–
Baseline AST level (IU/L) ^a^	22 (10–112)	56 (19–245)	41 (15–534)	64 (18–179)	–
Baseline ALT level (IU/L) ^a^	15 (7–113)	67 (19–314)	50 (9–820)	68 (14–201)	–
Baseline ALB (g/dL)	4.2 (3.2–4.9)	4.0 (3.0–5.0)	4.2 (3.0–5.1)	3.9 (3.3–4.5)	–
Baseline γ-GTP (IU/L)	31 (9–1511)	52 (11–227)	30 (9–263)	39 (17–197)	–
Baseline AFP (ng/mL)	5.3 (2–405)	9 (2.0–137.1)	4.8 (1.5–167)	11.3 (2.0–129.6)	–
Liver fibrosis stage (0/1/2/3/4/NA)	4/51/18/12/3/30	1/5/7/5/6/43	8/125/42/33/24/43	2/4/4/6/6/36	–
FIB-4 index ^a^	2.46 (0.39–9.2)	3.59 (1.3–9.1)	2.16 (0.3–9.3)	4.1 (1.6–17.3)	–
HCV genotype (1/2/NA)	47/69/2	33/14/20	179/82/13	37/16/5	–
Treatment protocol					
(PR, IFN + RBV/PegIFN, IFN/PI + PR)	107/8/3	36/24/7	218/27/29	37/16/5	–
Platelet count (´10^4^/µL) at SVR24 ^a^	17.0 (7.4–29.9)	16.2 (6.0–143)	18.2 (2.9–37.7)	14.4 (7.3–39.6)	–
AST level (IU/L) at SVR24^a^	22 (10–112)	28 (14–190)	22.0 (9–132)	25.5 (16–62)	–
ALT level (IU/L) at SVR24^a^	15 (7–113)	23 (10–168)	16.5 (3–130)	20.0 (6–68)	–
AFP at SVR24 (ng/mL)	3 (1–261)	5.5 (2–80)	3.4 (1.0–14.2)	4.70 (1.1–65.1)	–
FIB-4 index at SVR24^a^	1.93 (0.39–5.4)	1.86 (0.2–5.86)	1.83 (0.3–26.2)	2.61 (0.5–7.1)	–
Follow-up duration (month) after SVR	118 (24–283)	–	77 (30–330)	–	–
Median time to HCC occurrence (month)	–	48 (12–292)	–	40.0 (12–290)	–
f/u period after IFN completion (month)					
> 2 years	117		274		–
> 4 years	107		189		–
Genetic association:					
rs4778350 (chr15:24182473: G: A)					
(n (%))					
Genotype 1/1	54 (45.8)	15 (22.4)	113 (41.2)	17 (28.8)	–
Genotype 1/0	52 (44.1)	20 (29.9)	131 (47.8)	22 (37.3)	–
Genotype 0/0	12 (10.2)	32 (47.8)	30 (10.9)	20 (33.9)	–
Dominant model					
(0/0 vs 1/1 + 1/0)					
Combined	–	–	–	–	**5.86 (3.63–9.44)**
					***p*** **= 9.8× 10**^**− 15**^
GWAS stage	–	–	–	–	8.08 (3.76–17.4)
					*p* = 7.8× 10^− 9^
Replication stage	–	–	–	–	4.17 (2.16–8.06)
					*p* = 7.6× 10^− 6^

HCV, Hepatitis C virus; SNP, single nucleotide polymorphism; HCC, hepatocellular carcinoma; GWAS, genome-wide association study; chr, chromosome; MAF, minor allele frequency; rs, reference SNP; AFP, alpha fetoprotein; Alb, albumin; ALT, alanine transaminase; AST, aspartate aminotransferase; IFN, interferon; LC, liver cirrhosis

R-control, control group in replication cohort; R-HCC occurrence, HCC occurrence group in replication cohort

The major allele of each SNP is indicated by 1, whereas the minor allele by 0. *Odds ratio for the minor allele in a dominant model. ** *p*-value by the χ2 test for the minor allele dominant model

^a^ Data are shown as median values (range)

**Fig. 1:**
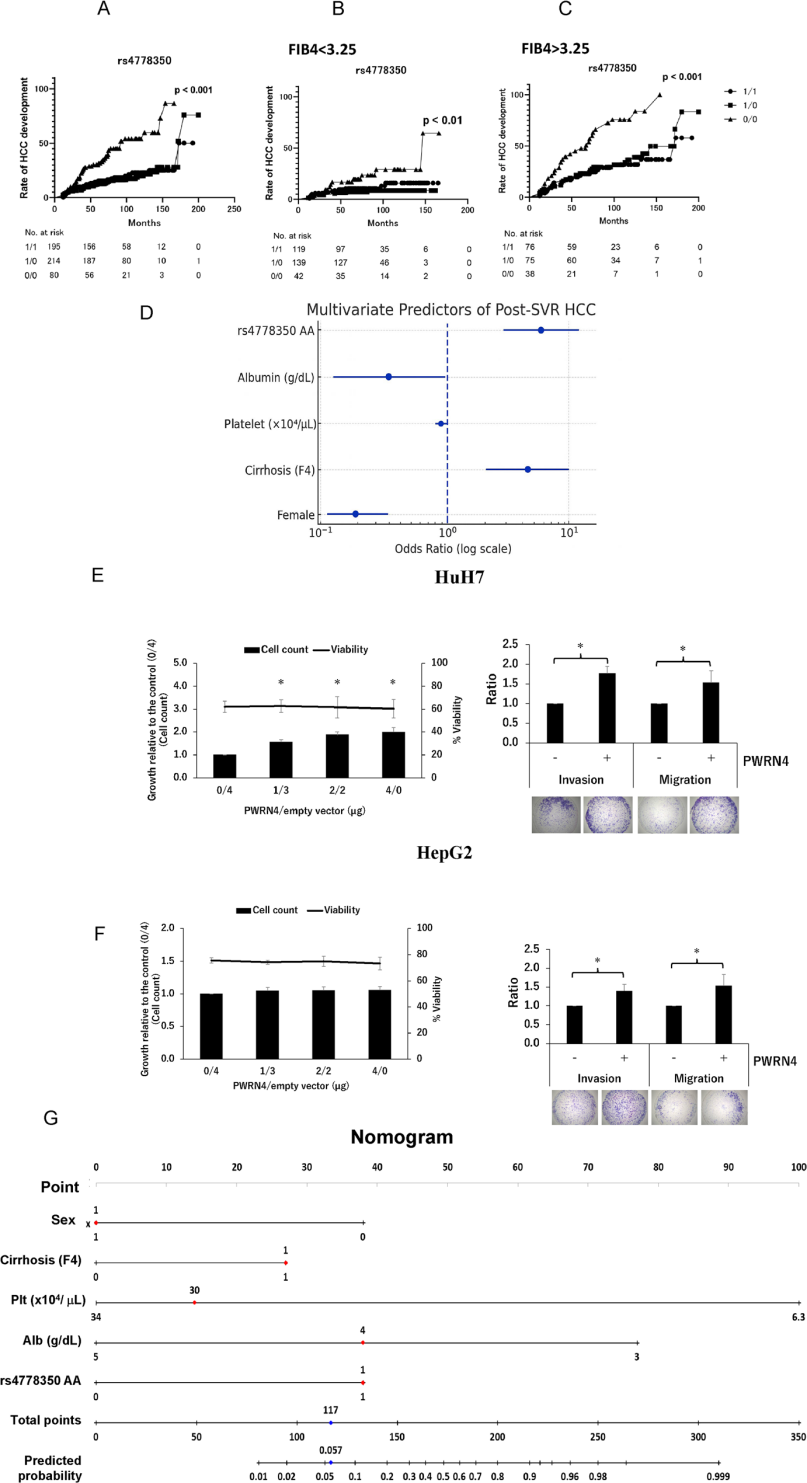
Cumulative incidence of hepatocellular carcinoma (HCC) occurrence after successful Hepatitis C virus (HCV) eradication by interferon (IFN)-based therapy, stratified by rs4778350 genotypes. The major allele of each SNP is designated ‘1’, and the minor allele ‘0’. Kaplan–Meier survival curves were compared using the log-rank (Mantel–Cox) test to assess the association of 1/1, 1/0, or 0/0 genotypes with HCC incidence. (**A**) Entire cohort. (**B**, **C**) Subgroup of patients with (**B**) or without (**C**) fibrosis (FIB-4 < 3.25; *p* < 0.01). (**D**) Multivariate predictors of HCC after sustained virologic response (SVR). Forest plot showing adjusted odds ratios (OR) and 95% confidence intervals (CIs) for five independent variables identified in the full cohort. Risk factors (OR > 1): Cirrhosis stage F4: OR 4.4 (95% CI, 2.0–9.6; *p* = 0.015), rs4778350 AA genotype: OR 5.6 (95% CI, 2.8–11.0; *p* < 0.001). Protective factors (OR < 1): Female sex: OR 0.22 (95% CI, 0.12–0.41; *p* < 0.001); Platelet count (per 1 × 10⁴/µL increase): OR 0.89 (95% CI, 0.80–0.99; *p* = 0.045); Serum albumin (per 1 g/dL increase): OR 0.33 (95% CI, 0.13–0.87; *p* = 0.026). (**E**, **F**) Functional impact of PWRN4 overexpression in hepatoma cells. Overexpression of PWRN4 in HuH7 cells (**E**) and HepG2 cells (**F**) increased proliferation (growth curve, left panels) and significantly enhanced cell migration and invasion (bar graphs, right panel) compared to that in control cells. These results support a pro-tumorigenic role of PWRN4 upregulation linked to the rs4778350 risk allele. (**G**) Nomogram for prediction of HCC development after SVR. The top horizontal axis (“Points”) assigns a score (0–100) to each predictor. To calculate total risk; draw a vertical line from the patient’s value on each variable axis to the “Points” axis, sum the scores on the “Total Points” axis, and project the total down to the “Predicted Probability” axis. Higher total points indicate a greater predicted probability of HCC development after SVR. Sex: 1 = female, 0 = male; Cirrhosis (F4): 1 = present, 0 = absent; Platelet (×10^4^ µL); Albumin (g/dL); Serum albumin concentration (continuous); rs4778350 AA: 1 = AA, 0 = the others

Mechanistically, the AA genotype of rs4778350 was associated with elevated hepatic expression of PWRN4, based on eQTL analysis (Supplementary Fig. S1b) [8]. PWRN4 is a primate-specific long non-coding RNA (lncRNA) whose function was previously unknown. Overexpression of PWRN4 in HuH7 (Fig. 1E) and HepG2 (Fig. 1F) hepatoma cells significantly enhanced cell proliferation, migration, and invasion in vitro. These findings suggest that the rs4778350 risk allele promotes hepatocarcinogenesis by upregulating a pro-tumorigenic lncRNA. Consistently, the 5-year HCC incidence was 29% in AA-genotype patients versus < 10% in those with GA/GG genotypes (*p* < 0.01) (Fig. 1A).

Notably, the minor (A) allele of rs4778350 exhibits substantial interethnic variation in frequency (Supplementary Fig. S2). It is considerably less common in East Asians (33.5% allele frequency) than in Europeans or Africans (59.4–63.8%). Consequently, the high-risk AA genotype is relatively rare (≈ 10% prevalence) in East Asia but more frequent in Western and African populations. This low prevalence may partly help explain why previous East Asian GWASs did not detect a significant association between rs4778350 and post-SVR HCC occurrence [7, 9, 10, 11]. The retrospective design, single-ethnicity cohorts, absence of key clinical covariates (e.g., alcohol intake and diabetes), and modest sample size limit generalizability, highlighting the need for prospective multi-ethnic validation. Additionally, because our analysis included only post-SVR patients, we could not assess whether rs4778350 predicts HCC in chronic hepatitis C, hepatitis B, or MASLD. This limitation highlights the need for future prospective studies. This study illustrates how a molecular epidemiologic approach combining GWAS and functional assays can reveal novel oncogenic pathways. We propose that PWRN4 and its associated variant rs4778350 constitute a novel biomarker axis for post-SVR HCC risk. To translate these findings into clinical practice, we have developed a risk score that combines rs4778350 with the other factors significantly associated with post-SVR hepatocarcinogenesis (Fig. 1D). The resulting nomogram is shown in Fig. 1G. While this tool may help identify high-risk patients, its predictive accuracy must be validated in larger, independent cohorts. Prospective validation in diverse populations will clarify its predictive value, while mechanistic studies may enable therapeutic targeting of PWRN4 to mitigate post-SVR hepatocarcinogenesis. Further studies are warranted to confirm the biomarker potential of rs4778350 and to explore therapeutic strategies targeting the PWRN4 pathway in hepatocarcinogenesis.

## Supplementary Information

Below is the link to the electronic supplementary material.

Supplementary Material 1

## Data Availability

The data that support the findings of this study are not publicly available due to privacy reasons but are available from the corresponding author upon request.

## Abbreviations

AFP: Alpha-fetoprotein
CI: Confidence interval
CT: Computed tomography
DAA: Direct-acting antiviral
eQTL: Expression quantitative trait locus
GWAS: Genome-wide association study
HCC: Hepatocellular carcinoma
HCV: Hepatitis C virus
IFN: Interferon
lncRNA: Long non-coding RNA
MAF: Minor allele frequency
MASLD: Metabolic dysfunction-associated steatotic liver disease
MRI: Magnetic resonance imaging
OR: Odds ratio
PWRN4: Prader–Willi region non-protein coding RNA 4
SNP: Single nucleotide polymorphism
SVR: Sustained virologic response
