# Supplementary material for: Long non-coding RNA PWRN4 associated with post-SVR hepatocellular carcinoma: a genome-wide association study
Source: Biomark Res. 2025 Sep 24;13:113. doi: 10.1186/s40364-025-00832-9 (PMC12462314; doi:10.1186/s40364-025-00832-9)
Supplement: Supplementary file 1 — Supplementary Material 1 [file 40364_2025_832_MOESM1_ESM.docx]

**Supplementary Information**

**Long non-coding RNA PWRN4 associated with post-SVR hepatocellular carcinoma: A genome-wide association study**

Goki Suda^a¶*^, Masaya Sugiyama^b¶^, Hayato Hikita^c^, Akira Nishio^c^, Tomohide Tatsumi^c^, Tetsuo Takehara^c^, Miyako Murakawa^d^, Mina Nakagawa^d^, Yasuhiro Asahina^d,p^, Masashi Mizokami^e^, Tatsuhiko Kakisaka^f^, Yuzuru Sakamoto^f^, Akinobu Taketomi^f^, Koji Miyanishi^g^, Yoshiyuki Ueno^h^, Hiroaki Haga^h^, Shinya Maekawa^i^, Nobuyuki Enomoto^i^, Masayuki Kurosaki^j^, Motoyuki Kohjima^k^, Makoto Nakamuta^k^, Yasuhito Tanaka^l^, Yoshiya Yamamoto^m^, Masaru Baba^n^, Hisatoshi Hanamatsu^o^, Jun-Ichi Furukawa^o^, Masatsugu Ohara^a^, Takashi Kitagataya^a^, Naoki Kawagishi^a^, Masato Nakai^a^, Takuya Sho^a^, Koji Ogawa^a^, and Naoya Sakamoto^a*^

**Methods**

**Patients and study design**

We collected genomic DNA samples from 118 patients who had not developed hepatocellular carcinoma (HCC) for at least two years after achieving a sustained virological response for 24 weeks after the end of treatment (SVR24) with IFN-based therapy and 67 patients who experienced HCC occurrence after successful HCV eradication between 2013 and 2018, as reported in 15 Japanese multicenter studies. In this study, only patients who developed HCC ≥12 months after achieving SVR were included; those with earlier onset were excluded. Subsequently, replication analysis was performed on genomic DNA samples from 15 institutions, including 274 patients with no HCC occurrence for ≥ 2 years after successful eradication by IFN-based therapy and 58 patients with HCC occurrence after successful eradication between 2018 and 2020.

Clinical data at baseline, including sex, age at the initiation of interferon (IFN)-based therapy, aspartate aminotransferase (AST), alanine transaminase (ALT), alpha-fetoprotein (AFP), HCV genotype, platelet count, albumin (ALB), liver fibrosis stage based on liver biopsy, Fibrosis-4 (FIB-4) index, and treatment protocols, were collected. Additionally, data on platelet count, AST, ALT, and FIB-4 index at SVR24, and the time to HCC occurrence after IFN-based therapy completion or follow-up duration to the last follow-up day without HCC occurrence were collected. Patients included in the study were evaluated for HCC occurrence every 3–6 months using ultrasonography (US), computed tomography (CT), or magnetic resonance imaging (MRI). Exclusion criteria included coinfection with HIV or HBV or both; other liver diseases; history of HCC; or decompensated liver cirrhosis.

The study protocol conformed to the ethical guidelines of the Declaration of Helsinki and the Declaration of Istanbul, and was approved by the Ethics Committee of Hokkaido University Hospital (approval number 16-040). All patients provided written informed consent before participating in the study. This study was registered with the UMIN Clinical Trials Registry (UMIN000031092). All authors had access to the study data and reviewed and approved the final manuscript.

**Anti-HCV protocols**

All patients with HCV were treated with IFN-based therapies, including 48 weeks of IFN monotherapy, 24–48 weeks of IFN plus ribavirin (RBV), 24–48 weeks of pegylated (Peg)-IFN plus RBV, 4–48 weeks of Peg-IFN monotherapy, or 24 weeks of Peg-IFN/RBV with simeprevir or telaprevir.

**Genome-wide association study (GWAS)**

A total of 185 genomic DNA samples from 67 Japanese patients with SVR who developed HCC and 118 patients without HCC for more than two years were assayed using the Illumina Infinium Japanese screening array, according to the manufacturer’s instructions. All 185 samples had an overall call rate > 97% and passed the heterozygosity check. The average overall call rate for the 185 samples was 99.51% (range: 97.23–99.53%).

Genotype imputation was performed on the filtered single nucleotide polymorphism (SNP) array data using BEAGLE 5.1 software [1]. Genotype data in VCF format were processed to match the reference panel using the conform-gt program, and imputation was performed using BEAGLE 5.1 with default settings. The reference panel for genotype imputation was made in-house and comprised 9338 haplotypes from 4669 individuals from diverse populations, including 2493 from the 1000 Genomes Project [2], 820 from the Human Genome Diversity Project [3], 278 from the Simons Genome Diversity Project [4], 90 from the Korean Personal Genome Diversity Project [5], and 1026 from Biobank Japan. The Biobank Japan data were approved controlled-access data obtained from NBDC (JGAS000114), while the other datasets were downloaded from public databases. Imputed variants (SNPs and indels) with low quality (DR^2^ < 0.5) were filtered, and genotypes were hard-called using the highest genotype probability; probabilities < 0.9 were considered no-calls.

For GWAS, only autosomal variants were analyzed; all sex-chromosome loci were excluded. Biological sex was inferred from the genomic data (X-chromosome heterozygosity and Y-chromosome markers) and entered as an independent covariate, separate from genotype, in the statistical models. Statistical associations between HCC development and genetic variants were tested using logistic regression. Variants with a minor allele frequency (MAF) < 1% or a low call rate < 95% were excluded from the analysis. Additive genetic effects were assumed. GWAS was conducted using PLINK 1.9 software.

**Replication of GWAS results**

To validate the GWAS data, 332 independent genomic DNA samples, from 58 Japanese patients with SVR who developed HCC and 274 with SVR and without HCC, were used for SNP typing using an in-house multiplex SNP assay (DigiTag2 method), according to the manufacturer’s instructions [6].

**Cell lines and transfection**

Human hepatoma (HuH7) and hepatoblastoma (HepG2) cell lines were obtained from the JCRB cell bank (Osaka, Japan). Cells were cultured in Dulbecco’s Modified Eagle Medium (DMEM; Invitrogen, Carlsbad, CA, USA) supplemented with 10% fetal bovine serum (FBS), 1% penicillin (100 U/mL), and streptomycin (100 μg/mL) (Gibco, Tokyo, Japan) in a humidified incubator at 37°C with 5% CO_2_. After 24 h, cells were transfected with pcDNA6/PWRN4 or pcDNA6 using lipofectamine 2000 (Invitrogen), according to the manufacturer's instructions. Briefly, on the day of transfection, the medium was removed and replaced with fresh serum-containing medium. Lipofectamine 2000/pcDNA complexes were then added to the wells. After 24 h, the transfection medium was replaced with fresh medium.

**Cell migration and invasion assays**

Approximately 3×10^4^ cells in 250 µL of serum-free DMEM were added to the upper chamber of each well. Fresh medium containing 10% FBS was then added to the lower chamber. For invasion assays, the inserts were incubated for 24 h using the same procedure described above, except that the wells were coated with 200 µg/mL matrigel. After 48 h of incubation, migrating and invaded cells in the lower chamber were stained with 0.1% crystal violet. Images of HuH7 cells were obtained under a microscope.

**Cell viability assay**

Cell proliferation was quantified using a Cell Counting Kit. Briefly, transfected cells were plated in 96-well plates at a density of 1.0×10^3^ cells per well in 100 μL and cultured for 24, 48, 72, and 96 h. Every 24 h, 10 mL of Cell-Counting Kit (CCK)-8 reagent (Dojindo, Kumamoto, Japan) was added to each plate. Absorbance was measured at 490 nm using a plate reader.

**Statistical analyses**

For GWAS analysis, the association between SNPs and HCC development was assessed using the χ^2^ test with a 2×2 contingency table in three genetic models: allele frequency, dominant-effect, and recessive-effect models. SNPs on the X chromosome were excluded because sex was not matched between the HCC and non-HCC groups. The significance levels for multiple testing were set at *p* = 5.0×10^−8^ in the GWAS stage. A total of 480 SNPs, comprising both significant and marginal levels, were selected for replication. Subsequently, a value of *p* = 1.0×10⁻⁴ (0.05/480) was defined for the replication stage. None of the markers genotyped in the replication stage showed deviations from Hardy–Weinberg equilibrium in our samples (*p* > 0.05).

Continuous variables were analyzed using the paired Mann–Whitney *U* test, Wilcoxon test, or one-way analysis of variance (ANOVA), as appropriate. Categorical data were compared using the Chi-squared test. Relationships between the two variables were assessed using Spearman’s rank correlation coefficients. Kaplan–Meier plots were used to compute survival curves, and the log-rank (Mantel–Cox) test was used to compare survival between groups. All variables were included in a multivariate logistic regression analysis to explain the association of variables with SVR-derived HCC. For nomogram development, time-to-event data were analyzed with a multivariable Cox proportional-hazards model using HCC occurrence after SVR as the endpoint. The five covariates identified in the discovery GWAS (sex, cirrhosis, platelet count, serum albumin, and rs4778350 genotype) were entered simultaneously without further selection. Each β-coefficient was divided by 0.25 and rounded to the nearest integer to generate a 0–100-point scale. These points were linearly mapped onto a nomogram axis; the resulting chart is shown in Fig. 1G. All *p*-values were two-tailed, and the level of significance was set to *p* < 0.05. All statistical analyses were performed using SPSS version 24.0 (IBM Japan, Tokyo, Japan).


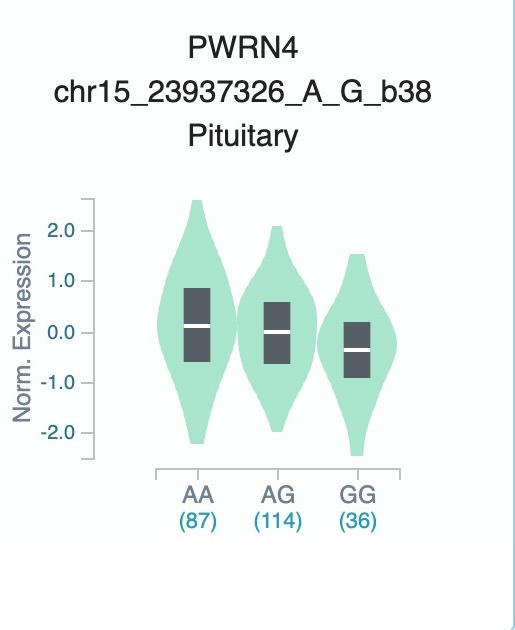


(**A**)


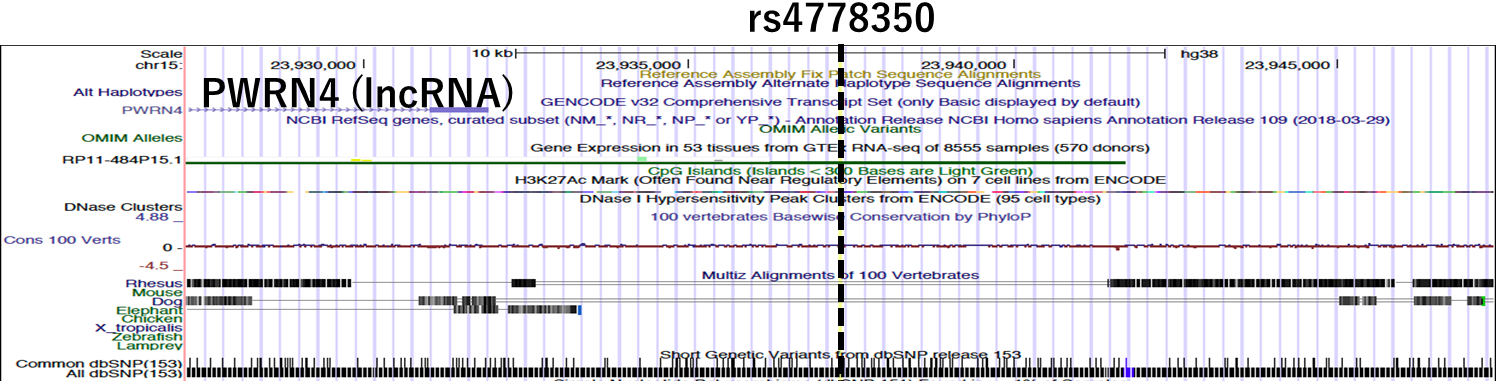
(**B**)

**Supplementary Fig S1.** Effect of rs4778350 on expression levels of the long non-coding RNA (lncRNA) Prader-Willi non-protein coding RNA 4 (PWRN4) based on expression quantitative trait locus (eQTL) analysis. (**A**) Genomic location of rs4778350, retrieved from the eQTL database. (**B**) Violin plot showing PWRN4 expression levels stratified by rs4778350 genotype, indicating a genotype-dependent regulatory effect on PWRN4 expression.

**(A)**


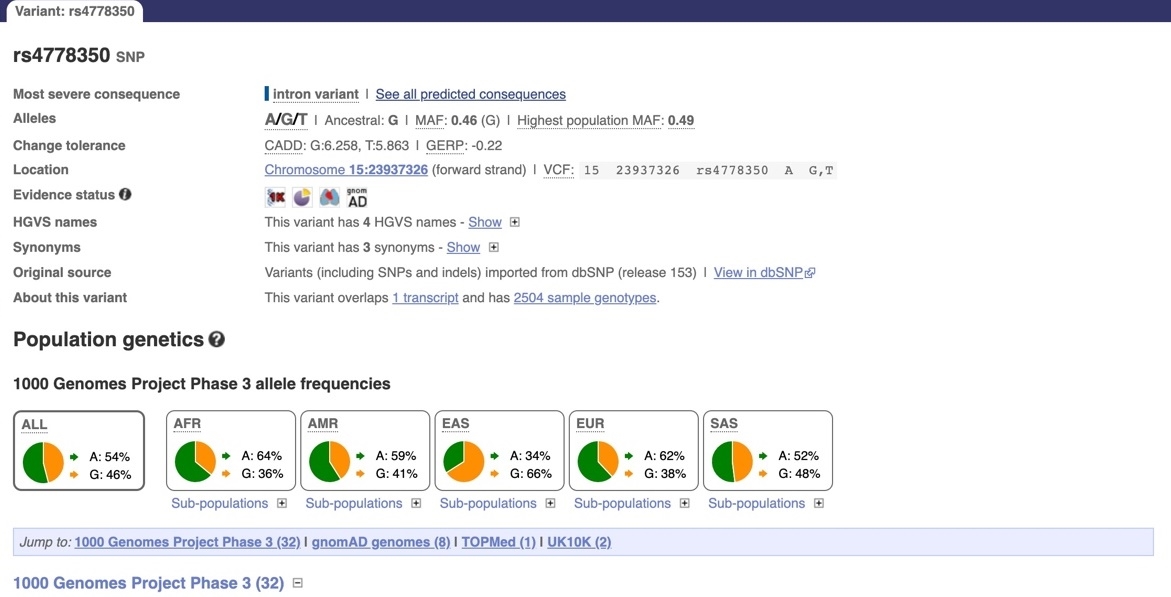


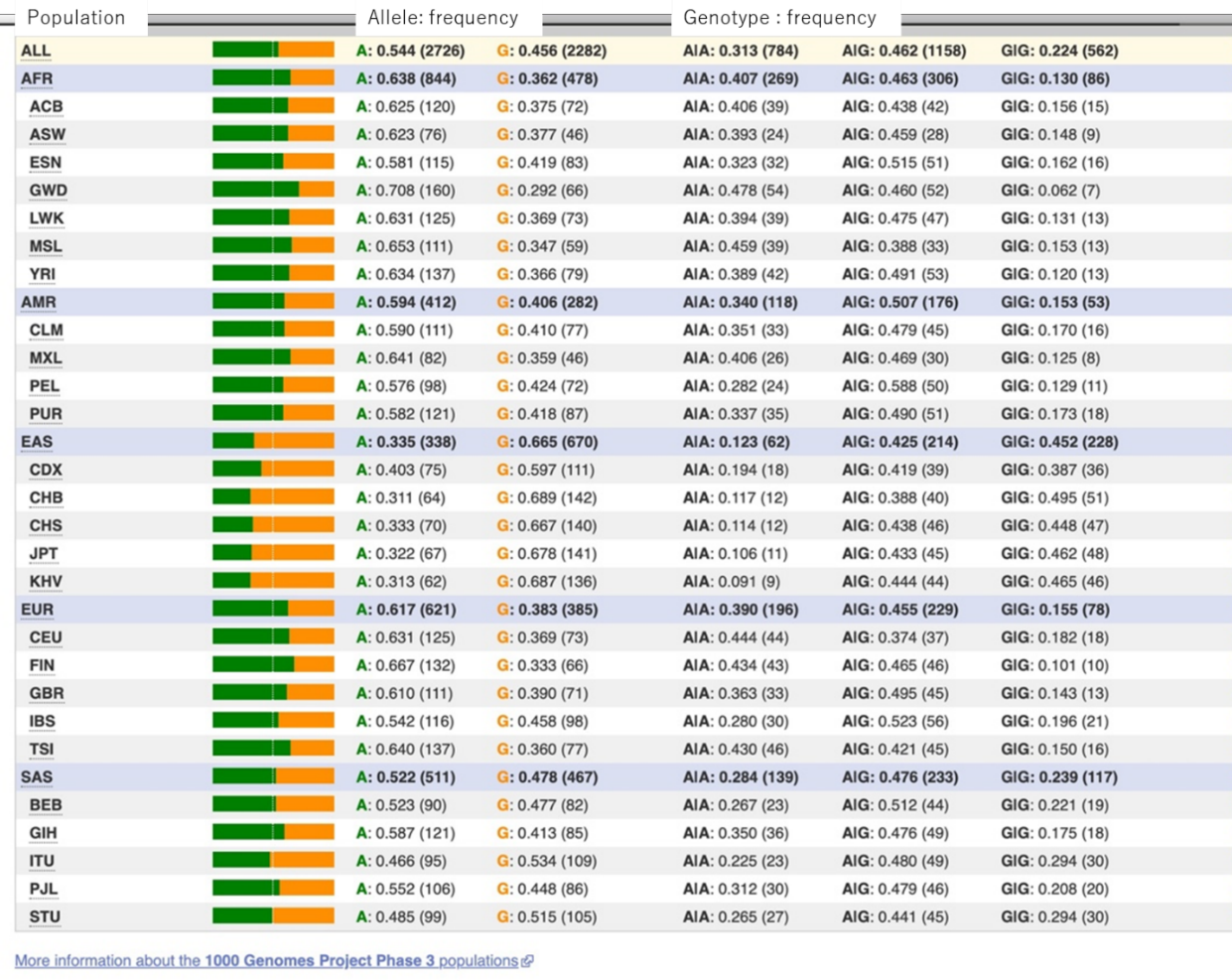


(**B**)

**Supplementary Fig S2.** Minor allele frequency (MAF) of rs4778350 in various ethnic groups. (**A**) Globally, the G allele is the minor allele of rs4778350, except in East Asia. (**B**) Regional allele frequency data for rs4778350, retrieved from the Ensembl genome browser. In East Asian populations, the A allele is the minor allele across all ethnic groups, contrasting with global trend.

**References**

1. Browning BL, Zhou Y, Browning SR: **A One-Penny Imputed Genome from Next-Generation Reference Panels**. *Am J Hum Genet* 2018, **103**(3):338-348.

2. Byrska-Bishop M, Evani US, Zhao X, Basile AO, Abel HJ, Regier AA, Corvelo A, Clarke WE, Musunuri R, Nagulapalli K *et al*: **High-coverage whole-genome sequencing of the expanded 1000 Genomes Project cohort including 602 trios**. *Cell* 2022, **185**(18):3426-3440 e3419.

3. Bergstrom A, McCarthy SA, Hui R, Almarri MA, Ayub Q, Danecek P, Chen Y, Felkel S, Hallast P, Kamm J *et al*: **Insights into human genetic variation and population history from 929 diverse genomes**. *Science* 2020, **367**(6484).

4. Mallick S, Li H, Lipson M, Mathieson I, Gymrek M, Racimo F, Zhao M, Chennagiri N, Nordenfelt S, Tandon A *et al*: **The Simons Genome Diversity Project: 300 genomes from 142 diverse populations**. *Nature* 2016, **538**(7624):201-206.

5. Kim J, Weber JA, Jho S, Jang J, Jun J, Cho YS, Kim HM, Kim H, Kim Y, Chung O *et al*: **KoVariome: Korean National Standard Reference Variome database of whole genomes with comprehensive SNV, indel, CNV, and SV analyses**. *Sci Rep* 2018, **8**(1):5677.

6. Nishida N, Mawatari Y, Sageshima M, Tokunaga K: **Highly parallel and short-acting amplification with locus-specific primers to detect single nucleotide polymorphisms by the DigiTag2 assay**. *PLoS One* 2012, **7**(1):e29967.
